# Association between Residential Proximity to PERC Dry Cleaning Establishments and Kidney Cancer in New York City

**DOI:** 10.1155/2009/183920

**Published:** 2010-01-24

**Authors:** Jing Ma, Lawrence Lessner, Judith Schreiber, David O. Carpenter

**Affiliations:** ^1^Department of Epidemiology and Biostatistics, University at Albany School of Public Health, Rensselaer, NY 12144, USA; ^2^Institute for Health and the Environment, University at Albany, Rensselaer, NY 12144, USA; ^3^New York State Office of the Attorney General, The Capitol, Albany, NY 12224-0341, USA

## Abstract

Perchloroethylene (PERC) is commonly used as a dry cleaning solvent and is believed to be a human carcinogen, with occupational exposure resulting in elevated rates of kidney cancer. Living near a dry cleaning facility using PERC has been demonstrated to increase the risk of PERC exposure throughout the building where the dry cleaning is conducted, and in nearby buildings. We designed this study to test the hypothesis that living in an area where there are many PERC dry cleaners increases PERC exposure and the risk of kidney cancer. We matched the diagnosis of kidney cancer from hospitalization discharge data in New York City for the years 1994–2004 by zip code of patient residence to the zip code density of dry cleaners using PERC, as a surrogate for residential exposure. We controlled for age, race, gender, and median household income. We found a significant association between the density of PERC dry cleaning establishments and the rate of hospital discharges that include a diagnosis of kidney cancer among persons 45 years of age and older living in New York City. The rate ratio increased by 10 to 27% for the populations in zip codes with higher density of PERC dry cleaners. Because our exposure assessment is inexact, we are likely underestimating the real association between exposure to PERC and rates of kidney cancer. Our results support the hypothesis that living near a dry cleaning facility using PERC increases the risk of PERC exposure and of developing kidney cancer. To our knowledge, this study is the first to demonstrate an association between residential PERC exposure and cancer risk.

## 1. Introduction

Perchloroethylene (PERC), also known as tetrachloroethylene or tetrachloroethene, is a volatile, nonflammable liquid with a sweet odor. It has been used as the primary solvent in the dry cleaning industry since the 1930s. Because of its volatility, PERC is released into the environment from processes used in dry cleaning establishments, by volatilization from dry cleaned clothing, from spills, and from wastes containing PERC such as still bottoms, waste water, used filters and spent carbon. The United States Environmental Protection Agency (EPA) estimates that there are about 34,000 dry cleaner facilities nationwide and approximately 82% of them use PERC as their primary solvent [1]. The Agency for Toxic Substances and Disease Registry [2] estimates that more than 650,000 workers might be regularly exposed to PERC [2]. Studies have shown that people exposed to PERC either by occupation or residential proximity to PERC dry cleaners have elevated levels of PERC in blood, exhaled breath, urine, and in breastmilk (in lactating women) [3–6]. The absorption and distribution of PERC to body tissues and fluids is not disputed, although adverse health effects of exposure are still being evaluated. 

Residential populations living close to dry cleaners are often exposed to levels of PERC considerably above background levels. Schreiber et al. [7] reported substantially elevated levels of PERC in apartments located above dry cleaning establishments (some as high as 55,000 micrograms per cubic meter, *μ*g/m^3^), as well as elevated levels of PERC in outdoor air near dry cleaners. These elevations were up to two orders of magnitude above those at distant sites, based on repeated 12-hour monitoring periods, both when the dry cleaners were operating and when they were not. In addition, next door neighbors' indoor air also shows elevated PERC levels, ranging from 11 (near background concentration) to 636 *μ*g/m^3^ [8]. 

PERC is classified as a hazardous air pollutant by the USEPA in the National Emission Standards for Hazardous Air Pollutants [1], and is considered “reasonably anticipated to be a human carcinogen” by the National Toxicology Program [9]. The International Agency for Research on Cancer classifies PERC as Group 2A, “probably carcinogenic to humans” [10]. PERC exposure has been related to development of kidney, bladder, liver, and esophageal cancer [9].

Occupational studies have demonstrated an association between PERC exposure and kidney cancer. Mandel et al. [11] investigated the relationship between occupational PERC exposure and kidney cancer in a study of 1732 cases and 2309 controls from Australia, Denmark, Germany, Sweden, and the United States, and found that exposure to dry cleaning solvents significantly increased relative risk (RR = 1.4; 95% CI, 1.1–1.7). McCredie and Stewart [12] analyzed risk of kidney cancer in New South Wales. They interviewed 489 cases of renal cell cancer, 147 cases of renal pelvic cancer, and 523 controls based on their employment in certain industries. Employment in the dry cleaning industry was strongly associated with both renal pelvic (RR = 4.68; 95% CI, 1.32–16.56) and renal cell (RR = 2.49; 95% CI 0.97–6.35) cancer. Katz and Jowett [13] evaluated at the mortality pattern of 671 female laundry and dry cleaning workers for the period of 1963–1977, using Wisconsin death certificate data. They found the standardized mortality odds ratio for developing kidney cancer was 2.57 (95% CI, 1.04–5.34). A similar study conducted by Duh and Asal [14] analyzed 330 laundry and dry cleaning worker for the period 1975–1981 using Oklahoma death certificate data. The standardized mortality odds ratio was calculated as 3.8 (95% CI, 1.48–7.59). Ulm et al. [15] conducted a meta-analysis of occupational studies of the association of kidney cancer and exposure to PERC, and reported a summary odds ratio of 1.49 (95% CI, 1.24–1.8).

**Figure 1:**
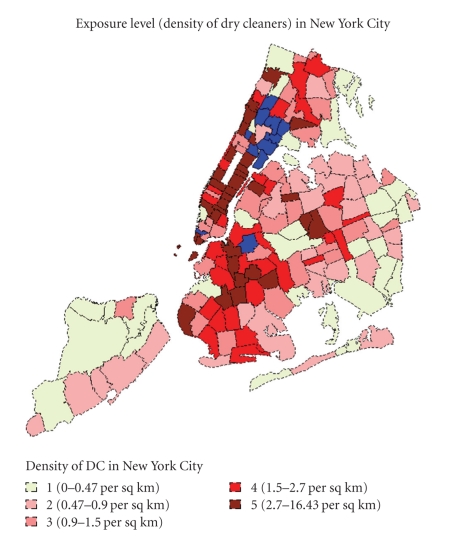
Map of dry cleaner density in New York City by zip code. Blue refers to excluded zip code.

We designed this study to test the hypothesis that living in an area where there are many PERC dry cleaners increases PERC exposure and the risk of kidney cancer. We used data available from the New York State Department of Health to assess by zip code the number of people hospitalized for treatment of kidney cancer (see Figure 1). We used the “density of dry cleaners that use PERC” by zip code in New York City as a surrogate for PERC exposure. We recognize that other solvents are also used in some of these dry cleaners, but the data from the registry documents that they use PERC, which was the criterion for inclusion.

Residential populations living close to PERC dry cleaning facilities are often exposed to levels of PERC considerably above PERC levels in buildings away from PERC dry cleaning facilities where background mean is 3 *μ*g/m^3^ [5]. Schreiber et al. [7] found that PERC levels were up to more than three orders of magnitude higher in residences located above PERC dry cleaning facilities than those at distant sites. While neurological effects (abnormalities in visual contrast sensitivity) can be detected in people following residential exposure to PERC [6, 16], there have been no investigations of cancer risks resulting from nonoccupational exposure to PERC. There is, however, reason to suspect that because of high indoor air PERC concentrations living very near a PERC dry cleaning facility might increase risk of cancer and other adverse effects of exposure. The National Emissions Standards for Hazardous Air Pollutants (NESHAP) EPA proposed rule reported cancer risk estimates as high as 3 in 100 (30,000 in one million) for residents exposed to PERC in indoor air based on exposure to a concentration of 5,000 *μ*g/m^3^. As a result of the high theoretical risks, the final NESHAP for Dry Cleaners [1] prohibits future colocation of dry cleaners in apartment buildings. 

There are about 900 small dry cleaning facilities using PERC in New York City, with about half located in buildings with residential tenants [17]. Based on the street address of PERC dry cleaning facilities in New York City, we determined that a large population is potentially exposed to PERC. We estimate that 105,250 persons live in buildings with PERC dry cleaning operations in New York City, including 10,500 children. Many more people live near these facilities, with approximately 2,269,000 people living within 200 meters of these facilities [18]. Others are exposed in buildings where PERC dry cleaners are co-located with offices, schools, medical facilities and other business, including strip malls [8].

## 2. Materials and Methods

The study population was all residents of New York City from 1993 to 2004 who were admitted as inpatients to a state-regulated hospital. Hospital discharge data were obtained from the New York Statewide Planning and Research Cooperative System (SPARCS) for the years 1993–2004. SPARCS requires all state-regulated hospitals to report to the NYSDOH the principal diagnosis and up to 14 other diagnoses for each inpatient upon discharge. Diagnoses are made according to the International Classification of Disease, Ninth Revision (ICD-9). The SPARCS data provides age, race, gender, and zip code of residence for each patient. In this study, we selected hospital discharge data with a diagnosis of kidney and/or renal cancer (ICD-9 189.0 and 189.1). 

The publicly available data includes only zip code of residence of the patient, not the hospital in which treatment was provided or information about possible occupational exposure. Since we do not have personal identifiers for SPARCS discharges, we are not able to distinguish multiple hospital discharges by a single individual from hospital discharges of distinct individuals. The outcome variable in this study is thus the frequency of disease diagnosis at hospital discharge by zip code of patient residence, not disease incidence. Incidence of kidney or renal cancer is not available from this dataset, which is neither a death nor a cancer registry. However in zip codes with increases in the rate of kidney cancer, assuming that that the access and willingness to use inpatient care are equal, we expect to see an increase in the rate of hospital discharges that include kidney cancer in the diagnostic codes. Furthermore the rate of diagnoses at discharge, although a novel measure, is an adequate and interpretable measure of the presence of disease, and may allow us to more easily detect a rare disease like kidney cancer.

Given the unknown latency between exposure and disease, it is possible that exposures prior to 1993 (the first year of data used) contribute to the cancers, but it is unlikely that the distribution of PERC dry cleaners is markedly different before 1993. New York State residents who seek out-of-state healthcare are not included in this dataset, nor are patients in federal hospitals, such as those operated by the Veterans Administration. In general the hospitalized population consists of individuals with relatively severe illness, such as the kidney and renal cancer studied here, and not individuals receiving outpatient or emergency room care. For New York City, the dataset contains about 800,000 discharges per year for twelve years. With this dataset we can track residence near PERC facilities, but not the duration of residential or occupational exposure to PERC.

We studied only those zip codes where the median household income fell in the range from $17,864 to $142,926. Zip codes outside of this range were excluded. This restriction criterion was selected based on evidence [19–21] that rates and causes of hospitalization for individuals at both extremes of income are quite different from those in the group selected. Of the total of 181 zip codes in New York City, six zip codes were not considered because of having no population or income information (these are post office box zip codes), and ten zip codes were not considered because they did not meet the inclusion criteria (1 zip code had median household income greater than $142,926 and 9 zip codes below $17,864). The analysis was then based on the remaining 164 zip codes. 

Zip code-level population data was derived from US Census data, obtained from Claritas, Inc. (http://www.claritis.com/eReports/default.jsp) which provides population totals for each zip code stratified by age, race, and gender. We selected our study population of persons at least 45 years old, because kidney and renal cancer are rare in younger persons, and to account for the expected latency period between exposure and disease as well as the general decrease in use of PERC over time. Age was further divided into four groups: 45 to 54, 55 to 64, 65 to 74, and, 75 years and above, and restricted to Caucasian (white) and African American (black) populations due to the large number of individuals in these groups. We used Claritas zip code-level median household income information to control for socioeconomic status. Zip code level median household income is divided into three approximately equal groups: $17,864 to $33,353; $33,354 to $48,996; $48,997 to $142,926. Population density was also derived from Claritas data and was defined as the number of persons per square km, and was similarly divided into three approximately equal groups: 480 to 6,671, 6,672 to 17,509, and 17,510 to 60,102 persons per square km. 

By federal law and New York State regulation, each dry cleaner is required to report its usage of PERC. We used the list of dry cleaners using PERC which is maintained by the New York State Department of Environmental Conservation to determine the density of dry cleaners that use PERC in each zip code in New York City (the number of dry cleaners using PERC in a zip code divided by area of the zip code). In the absence of measurements of PERC concentrations at all sites, we use the density of dry cleaners using PERC in each zip code as a surrogate measure of PERC exposure. We did not incorporate information on the volume of PERC used, as this varies year by year and in general has declined over time because of increasing regulatory standards after 1996. The density of dry cleaners was divided into five equal groups by zip code based on the assumption that a higher dry cleaner density in a zip code leads to greater PERC exposure for persons living in that zip code. The highest density of dry cleaners was in Manhattan and some areas of Queens. See Table 1 for the distribution of density of PERC dry cleaners' exposure levels used in our analysis.

The unit of analysis is the population living in a zip code. These subpopulations were defined by the strata formed from the variables age, race, gender, population density, and median household income within each zip code. The outcome measured was the number of hospital discharges for each zip code population with a principal or other diagnosis of kidney cancer. We used a log linear model for hospital discharge rate, regressed on exposure and other covariates as follows: 

Log (expected number of kidney cancer discharges) = log {(person time) + Intercept + b1 ∗ Exposure 2 + b2 ∗ Exposure 3 + b3 ∗ Exposure 4 + b4 ∗ Exposure 5 + b6 ∗ middle population density + b7 ∗ High population density + b8 ∗ middle MHI + b9 ∗ High MHI + b10 ∗ Age 55–64 + b11 ∗ Age 65–74 + b12 ∗ Age 75 and above + b13 ∗ Black + b14 ∗ Female + Interactions}.

Initially we used a Poisson distributed log linear model, which resulted in a deviance/degree of freedom of 2.0, indicating over-dispersion and a poor fit. We therefore applied a negative binomial model which resulted in a deviance/degree of freedom of 1.25, indicating an adequate fit. 

Because the distribution of the covariates of the populations living in different zip codes is not the same, the model was examined for effect modifiers in the exposure versus outcome association. In particular, all interactions at each exposure level with age, gender, race, population density and median household income were considered. The deviance/dF for the model remained unchanged, indicating adequate fit. A residual analysis was conducted on the final model in order to determine whether any one or more zip codes exerted excessive leverage. We excluded each zip code, one at a time, and checked if the parameter estimates and the estimated rate ratios changed significantly. Three observations were readily identified as extreme values in Exposure levels 1 and 2 (the lowest and second lowest exposure levels), and when they were included in the analysis we found differences of as much as 20% in the estimated regression coefficients. In each of these observations the observed number of hospital discharges was much smaller than the expected number. This extraordinary dependence on a few observations is considered undesirable. Consequently these three observations were dropped from the analysis. The resulting analysis fit well, residuals looked good and there were no other observations with extraordinary leverage on the estimated parameters. 

All statistical analysis was performed with SAS software, Version 9.1 (SAS Institute Inc.).

## 3. Results

Table 2 presents information on all cancer discharges, including or excluding skin cancers, and all kidney and renal cancers by exposure category. Our analysis found no significant relationship between the density of PERC dry cleaners and “all cancer,” but, more importantly, did identify a significant association between the density of PERC dry cleaning establishments and the rate of hospital discharges that included a diagnosis of kidney cancer (ICD9 189.0 and 189.1). Table 3 presents results that employ a main-effects- only model and presents rate ratios and comparisons of the rate of kidney discharges of a given strata with the baseline strata for exposure as well as the other risk factors. Exposure levels 2, 4 and 5 are all positive, statistically significant, and quite similar, with rate ratios (RR) of 1.14, 1.17, and 1.15, respectively, and with *P*-values of .01, .006, and .03, respectively. Exposure level 3 is marginally significant with a *P*-value of .15. The estimated rate ratios for age are positive, statistically significant, and increase monotonically. The discharge diagnosis rate is higher for males than females. This is consistent with the evidence that age and gender are important risk factors for kidney cancer. The discharge rate is larger for Caucasians than for African-Americans. This pattern is consistent with the crude hospital discharge rate for NYC from 1993 to 2004, which shows white males with the highest discharge rate, followed by black males, white females, and black females (see Table 3).

Table 4 presents the estimated rate ratios and their confidence intervals from the main effects and interactions model, with information on effect modifiers particular to each exposure category. Rate ratios compare the effect in Exposure level 1 with the effect in Exposure levels 2, 3, and 4, and, are summary estimates obtained from weighted averages that will be discussed later. For example, the rate ratio for the entire population in Exposure level 4 was 1.27, while for the white population it was 1.35. This means that the rate ratio for kidney cancer in whites, living in Exposure level 4 versus Exposure level 1 increased by 35% (statistically significant). For the same comparison, the rate ratio for kidney cancer in blacks was 1.08, indicating an increase of 8% (not statistically significant). This difference with race might reflect genetic sensitivity as well as frequency of use of dry cleaners, although race was not found to be an effect modifier in the other exposure levels.

 We found that for each exposure level, the effect modifiers were different. We found no effect modifier for Exposure level 2. But for Exposure level 3, population density was an effect modifier, and the middle and high population density levels were statistically significant interactions. Race was an effect modifier only in Exposure level 4. Exposure level 5 had two effect modifiers, median household income and age. Within the low and medium household income group, there was an increase in the rate ratio with age. In the high income group there was a substantial increase in the rate ratio beginning at an earlier age, which indicates greater risk. Increases in rates of disease with advancing age are to be expected, but here the interaction of median household income with age is a more complex effect modification than that of age alone. We speculate that the higher income implies a greater use of dry cleaners.

 In order to obtain a summary rate ratio for the effects of exposure at each exposure level, we exponentiated the weighted average of the beta coefficients. For example, in Exposure level 3, population density is the effect modifier. The beta coefficient for low population density is −0.12, while for both middle and high population density it was 0.15. Using a standard population composition in NYC (low density, 19.6%; middle density, 43%; high density, 37.4%), we obtained a weighted average, −.12(.196) +.15(.43) +.15(.374) = .098 for a log-linear estimate. The summary rate ratio is exp (.098) = 1.102. The summary rate ratios calculated this way are comparable across different exposure levels. The rate ratios for Exposure levels 2 to 5 are all positive, ranging from 1.10 to 1.27. Thus, we see an increase from 10 to 27% in the rate of discharges for Exposure levels 2 to 5 compared to Exposure level 1.

In order to validate our choice of exposure classification, we conducted a permutation test. The 164 zip codes in the study were randomly assigned into five exposure levels and the same main effect model was applied. Then we examined the sign of the exposure coefficients and the significance of those coefficients. In a sample of 5000 iterations, the probability of getting an analysis with positive and statistically significant (*P*-value <.05) coefficients for Exposure levels 2, 4, and 5 was very low, 1.42%. Because each random assignment or iteration is equivalent to a different exposure measurement, this permutation test indicated that using density of PERC dry cleaners as a measure of exposure was critical to finding a significant association with the rate of hospital discharge that included a diagnosis of kidney cancer.

## 4. Discussion

We found a significant association between the density of dry cleaning establishments using PERC and the rate of hospital discharges that include a diagnosis of kidney cancer among persons 45 years of age and older living in New York City. The rate ratio increased by 10 to 27% for the populations living in zip codes with higher density of PERC dry cleaners (i.e., Exposure levels 2, 3, 4, and 5 compared to Exposure level 1). These observations are consistent with the hypotheses that living near to a PERC dry cleaning establishment increases the risk of exposure to PERC, and that increased exposure to PERC increases the risk of developing kidney cancer. These results are compelling because of their strength of association and consistency with animal and occupational studies, particularly in light of the limitations in exposure assessment in this study. Other studies of residential populations have demonstrated that people living in such buildings are exposed to PERC and are at risk of neurological effects [3, 6, 16]. 

Effect modifiers, age, and MHI, in Exposure level 5, reflect the general trend toward greater exposure and risk for males (predominately white) with an increased risk as income and age increase, possibly because of greater use of dry cleaning, greater likelihood of living in or near a building with a dry cleaner, or living in an area of higher population density which implies more exposure to PERC per person. The elevated risk found at younger ages in the high income zip codes is particularly striking.

## 5. Limitations

There are several significant limitations in our study. Population density is based on the subpopulation of a zip code and can vary over time. Dry cleaner density per zip code is a crude measure of PERC exposure. A more satisfactory approach would include the individual level of exposure and the distance from a dry cleaning establishment to the residence, but information on the residential address of patients was not available. 

Dry cleaning facilities use differing amounts of PERC and have differences in emission controls, and operate in buildings with differing structures, building integrity, ventilation, and other variables which are not incorporated in our model. Air currents may influence the concentration of PERC in a zip code as well. Using density of PERC dry cleaners as a measure of exposure does not capture all aspects of residential exposure. For example, one would expect exposure to be greater in apartments co-located with PERC dry cleaners, and to decrease in buildings nearby with increasing distance. 

Zip codes in New York City, in general, are smaller in area than in other regions having lower population density. Thus exposure may be more uniform in a small area. We used all zip codes that met our inclusion criteria, including those in Staten Island which cover large areas and have relatively low population density leading to a large variation in PERC exposure within that zip code. We have no information on population mobility, and only know current zip code of residence. We have no direct measurements of PERC levels in the residences of patients or controls. In addition we have no information concerning occupational exposures. All of these limitations serve to attenuate the measure of effect, the rate ratio. 

In spite of these limitations our study has significant strengths. The SPARCS data is comprehensive for more than 10 years, and the large number of hospitalizations that include kidney cancer as well as our outcome variable, rate of discharges, allows detection of patterns of disease distribution that would otherwise not be discernable. We have detailed information on the location and use of PERC by dry cleaning establishments. We also have demonstrated that the results cannot be explained by random zip code assignments.

## 6. Conclusions

Given the significant limitations in our study, particularly in exposure assessment, the relationship between residential exposure to PERC and kidney cancer may be stronger than what we report in this study. This suggestion is consistent with the cancer risk estimate made previously by EPA (3 in 100 residents in buildings containing a PERC dry cleaning establishment). Clearly further study of the relationship between residential exposure to PERC and kidney cancer is needed, with more rigorous exposure and cancer risk assessment. Ideally a study comparing residents living in buildings with PERC dry cleaning establishments to residents far from PERC dry cleaners should be conducted. 

The analysis presented here supports the hypothesis that residential proximity to PERC dry cleaning facilities in New York City increases the risk of kidney cancer. Because a large residential population is potentially exposed to PERC from these facilities, more evaluation is needed to determine whether such a relationship would be found in a cross-sectional or case-control study.

## Figures and Tables

**Table 1:** Summary of exposure levels based on density of dry cleaners.

Exposure level	Density of dry cleaners
	(dry cleaners per sq km)
Exposure level 1	0 to 0.47
Exposure level 2	0.47 to 0.90
Exposure level 3	0.90 to 1.50
Exposure level 4	1.50 to 2.70
Exposure level 5	2.70 to 16.43

**Table 2:** Total cancers by exposure levels.

Exposure	All cancer	All cancer	Kidney/renal
	(exc. skin cancer)	(Inc. skin cancer)	cancer
1	90,908	91,510	1,458
2	131,165	132,014	2,289
3	113,629	114,396	1,838
4	162,945	164,093	2,766
5	171,170	172,506	2,565
Total	669,817	674,519	10,916

**Table 3:** Rate ratios for exposure levels and covariates from the main effects only model.

Variable	Rate ratio	95% CI	
By Exposure level			
Exposure level 1	1.00		
Exposure level 2	1.14	1.03	1.27
Exposure level 3	1.09	0.97	1.21
Exposure level 4	1.17	1.05	1.32
Exposure level 5	1.15	1.01	1.30

By population density			

Low	1.00		
Middle	0.84	0.76	0.93
High	0.66	0.59	0.74

By median household income (MHI)			

Low	1.00		
Middle	0.96	0.89	1.04
High	1.04	0.95	1.14

By age group			

Age 45–54	1.00		
Age 55–64	2.22	2.02	2.44
Age 65–74	3.77	3.44	4.14
Age 75+	4.22	3.85	4.64

By race			

Caucasian (white)	1.00		
African American (black)	0.89	0.83	0.95

By gender			

Male	1.00		
Female	0.44	0.41	0.47

**Table 4:** Rate ratios for exposure levels and interactions of effect modifiers.

	Rate ratio all population	Effect modifier	Effect modifier level	Rate ratio (RR) for certain level effect modifier	95% confidence interval for RR	
Exposure level 1	1	None				
Exposure level 2	1.15*	None				
	(95% CI 1.04, 1.27)					

Exposure level 3	1.102	Population density	Low	0.90	0.74	1.08
	(95% CI 1.00, 1.24)		Middle	1.16	1.01	1.33
			High	1.19	1.01	1.41

Exposure level 4	1.27*	Race	White	1.35	1.19	1.53
	(95% CI 1.13, 1.42)		Black	1.08	0.83	1.40

Exposure level 5			Low/mid MHI			
			Age = 45–54	0.87	0.70	1.07
			Age = 55–64	1.20	0.94	1.53
			Age = 65–74	1.21	1.00	1.46
	1.16*	MHI and Age	Age = 75 +	1.24	1.04	1.49
	(95% CI 1.02, 1.33)		High MHI			
			Age = 45–54	1.14	0.87	1.48
			Age = 55–64	1.57	1.18	2.09
			Age = 65–74	1.59	1.24	2.03
			Age = 75 +	1.63	1.28	2.07

*Statistically significant at *P* < .05.
